# 24-h PCI model does affect the outcome of STEMI patients: a population-based study

**DOI:** 10.1038/s41598-023-40276-5

**Published:** 2023-08-11

**Authors:** Chang-Hung Tsai, Pei-Tseng Kung, Shun-Mu Wang, Tung-Han Tsai, Wen-Chen Tsai

**Affiliations:** 1 Miao-Li General Hospital, Ministry of Health and Welfare, Miaoli, Taiwan, R.O.C.; 2 Department of Health Services Administration, China Medical University, No. 100, Section 1, Jingmao Road, Beitun District, Taichung City, 406040 Taiwan, R.O.C.; 3 Department of Public Health, China Medical University, Taichung City, Taiwan, R.O.C.; 4 Department of Senior Services Industry Management, Minghsin University of Science and Technology, Hsinchu, Taiwan, R.O.C.; 5 Department of Healthcare Administration, Asia University, Taichung City, Taiwan, R.O.C.; 6 Department of Medical Research, China Medical University Hospital, China Medical University, Taichung City, Taiwan, R.O.C.

**Keywords:** Cardiovascular diseases, Health policy, Health services, Cardiology, Health care, Medical research

## Abstract

Acute myocardial infarction has been the second leading cause of death in Taiwan. It’s a novel issue to evaluate the relationship between the 24-h PCI service model and the outcome of STEMI patients. The objective of this study was to determine the effect of 24-h PCI service model in STEMI patients to improving survival rate. This population-based cohort study included those STEMI patients, older than 18 year-old, who had ever called emergency department from 2012 to 2018. We had two groups of our study participant, one group for STEMI patients with 24-h PCI model and the other group for STEMI patients with non-24-h PCI model. We used the Logistic regression model to analyze the risk of death within 30 days, emergency department (ED) revisits within 3 days, and readmission within 14 days. After the relevant variables were controlled, the risk of death after an ED visit among the patients with STEMI who were sent to hospitals with 24-h PCI services was significantly lower than that among the patients with STEMI who were sent to hospitals without 24-h PCI services (OR 0.85; 95% CI 0.75–0.98). However, the model could not reduce the risk of ER revisits and readmission.

## Introduction

Acute myocardial infarction (AMI) is the second leading cause of death in Taiwan. In 2021, the death toll due to AMI reached 21,852, and an average death toll was 45.6 per 100,000 people^1^. In the United States, approximately 550,000 patients are newly diagnosed with myocardial infarction (MI) each year, and approximately 200,000 patients experience the recurrence of MI^2^. A 2017 study recommended that patients with ST elevation myocardial infarction (STEMI) should be treated with percutaneous coronary intervention (PCI) within 90 min after arriving at the emergency department of a hospital that can perform 24-h PCI.

A hospital revisit that occurs shortly after an emergency department visit (usually defined as a visit made within 72 h [3 days]) is referred to as an early return visit. The early return rate is regarded as a key indicator of the quality of emergency care^3–6^. Studies have reported that patients who made early return visits had a higher mortality rate^6^ and higher risk of being involved in medical disputes^4^. A Taiwanese study indicated that the rate of emergency department revisits within 3 days after discharge was approximately 5.47% and that the major reason for revisits was disease-related factors (80.9%), followed by patient-related factors (10.9%), and then doctor-related factors (8.2%)^7^. Therefore, the rate of hospital revisits within 3 days after discharge is a key indicator of the quality of emergency care, and it also affects patient prognosis and subsequent medical cost.

According to U.S. statistics pertaining to the period from 2010 to 2014, the rate of hospital readmission within 30 days after discharge among patients with STEMI was 12.3%^8^. Another large-scale, nationwide U.S. study revealed that 10.3% of patients with STEMI who had multiple coronary arteries unblocked were hospitalized again within 30 days after discharge, and within this subgroup of patients with STEMI, 62.66% were hospitalized because of cardiac factors^9^. Furthermore, women, patients with a family history of coronary artery disease, patients with a low family income, and patients who were treated in hospitals located in highly urbanized areas or nonteaching hospitals were reported to have a significantly higher rate of hospital readmission within 30 days after discharge relative to other groups of patients^8^. Patients with STEMI who were readmitted within 30 days after their emergency department visits were verified to have a significantly higher mortality rate (increase of 42%)^10^.

At present, not all hospitals in Taiwan provide 24-h PCI services. The PCI services in Taiwan can be divided into two models, namely the 24-h PCI service model and the non-24-h PCI service model. In the present study, we explored whether these models significantly affect patients with STEMI in terms of their risk of death within 30 days after emergency department visits, emergency department revisits within 3 days after discharge, and readmissions within 14 days after discharge from an emergency department.

## Results

Overall, 27,219 patients with STEMI were initially included; however, 231 patients aged less than 18 years, 3160 who had been diagnosed with STEMI, 89 who died of accidents, and 3091 who were not hospitalized (patients with STEMI should be hospitalized in Taiwan) were excluded. Eventually, 20,648 patients with STEMI were selected as participants.

After a chi-square test was conducted, a bivariate analysis was performed to determine whether patients with STEMI died within 30 days after their emergency department visits, the patients with STEMI revisited the emergency department within 3 days after they were discharged from an emergency department, and the patients with STEMI were readmitted within 14 days after their discharge from an emergency department. Table 1 reveals that 2281 patients with STEMI (11.05%) died within 30 days after an emergency department visit; among the patients who were sent to hospitals with a 24-h PCI service model, the mortality rate within 30 days after an emergency department visit was 7.71%, which was lower than that of patients who were sent to hospitals without a 24-h PCI service model (12.12%; *P* < 0.05). Table 1 also reveals that 234 patients with STEMI (1.13%) revisited an emergency department within 3 days after discharge. The rate of emergency department revisits within 3 days after discharge among the patients who were sent to hospitals with a 24-h PCI service model was 0.58%, which was significantly lower than that among the patients who were sent to hospitals without a 24-h PCI service model (1.31%; *P* < 0.05). Table 1 reveals that 246 patients with STEMI (1.19%) were readmitted within 14 days after discharge from an emergency department. Among the patients who were treated in hospitals with a 24-h PCI service model, the rate of readmission within 14 days after discharge from an emergency department was 0.92%, which was significantly lower than that among the patients who were treated in hospitals without a 24-h PCI service model (1.28%; *P* < 0.05).

**Table 1:** Bivariate analysis of the relationship between PCI service model and death within 30 days after emergency department visit, the rate of emergency department revisits within 3 days, and the rate of readmission within 14 days among the patients with STEMI.

Variables	Total		Death within 30 days					Emergency department revisits within 3 days					Readmission within 14 days				
			No		Yes		*p*-value	No		Yes		*p*-value	No		Yes		*p*-value
	N	%	N	%	N	%		N	%	N	%		N	%	N	%	
Total number	20,648	100.00	18,367	88.95	2,281	11.05		20,414	98.87	234	1.13		20,402	98.81	246	1.19	
PCI service model							< 0.001					< 0.001					0.043
Non-24-h	15,628	75.69	13,734	87.88	1,894	12.12		15,423	98.69	205	1.31		15,428	98.72	200	1.28	
24-h	5,020	24.31	4,633	92.29	387	7.71		4,991	99.42	29	0.58		4,974	99.08	46	0.92	
Gender							< 0.001					0.009					0.510
Female	3,846	18.63	3,066	79.72	780	20.28		3,787	98.47	59	1.53		3,796	98.70	50	1.30	
Male	16,802	81.37	15,301	91.07	1,501	8.93		16,627	98.96	175	1.04		16,606	98.83	196	1.17	
Age							< 0.001					< 0.001					0.007
< 45	2,084	10.09	2,011	96.50	73	3.50		2,071	99.38	13	0.62		2,067	99.18	17	0.82	
45–54	4,277	20.71	4,084	95.49	193	4.51		4,241	99.16	36	0.84		4,239	99.11	38	0.89	
55–64	5,578	27.01	5,242	93.98	336	6.02		5,527	99.09	51	0.91		5,512	98.82	66	1.18	
65–74	4,135	20.03	3,659	88.49	476	11.51		4,091	98.94	44	1.06		4,084	98.77	51	1.23	
75–84	3,146	15.24	2,469	78.48	677	21.52		3,094	98.35	52	1.65		3,101	98.57	45	1.43	
≧ 85	1,428	6.92	902	63.17	526	36.83		1,390	97.34	38	2.66		1,399	97.97	29	2.03	
Mean ± SD	62.95 ± 14.08		61.64 ± 13.55		73.55 ± 13.79			62.89 ± 14.07		68.34 ± 14.68			62.91 ± 14.08		66.49 ± 14.24		
CCI score							< 0.001					< 0.001					< 0.001
0	7,656	37.08	7,211	94.19	445	5.81		7,599	99.26	57	0.74		7,593	99.18	63	0.82	
1	6,039	29.25	5,447	90.20	592	9.80		5,973	98.91	66	1.09		5,967	98.81	72	1.19	
2	2,492	12.07	2,128	85.39	364	14.61		2,454	98.48	38	1.52		2,460	98.72	32	1.28	
3	1,819	8.81	1,534	84.33	285	15.67		1,793	98.57	26	1.43		1,795	98.68	24	1.32	
≧ 4	2,642	12.80	2,047	77.48	595	22.52		2,595	98.22	47	1.78		2,587	97.92	55	2.08	
Monthly salary (NTD)							< 0.001					0.349					0.011
≦ 17,280	5,257	25.46	4,503	85.66	754	14.34		5,186	98.65	71	1.35		5,190	98.73	67	1.27	
17,281–22,080	3,176	15.38	2,770	87.22	406	12.78		3,145	99.02	31	0.98		3,138	98.80	38	1.20	
22,081–36,300	7,725	37.41	6,946	89.92	779	10.08		7,640	98.90	85	1.10		7,617	98.60	108	1.40	
≧ 36,301	4,490	21.75	4,148	92.38	342	7.62		4,443	98.95	47	1.05		4,457	99.27	33	0.73	
Urbanization							< 0.001					0.001					0.001
Level 1	4,287	20.76	3,877	90.44	410	9.56		4,253	99.21	34	0.79		4,262	99.42	25	0.58	
Level 2	6,483	31.40	5,806	89.56	677	10.44		6,419	99.01	64	0.99		6,399	98.70	84	1.30	
Level 3	3,913	18.95	3,552	90.77	361	9.23		3,871	98.93	42	1.07		3,862	98.70	51	1.30	
Level 4	3,355	16.25	2,898	86.38	457	13.62		3,308	98.60	47	1.40		3,309	98.63	46	1.37	
Level 5–7	2,610	12.64	2,234	85.59	376	14.41		2,563	98.20	47	1.80		2,570	98.47	40	1.53	
Other catastrophic illness							< 0.001					0.001					0.019
No	19,137	92.68	17,194	89.85	1,943	10.15		18,933	98.93	204	1.07		18,919	98.86	218	1.14	
Yes	1,511	7.32	1,173	77.63	338	22.37		1,481	98.01	30	1.99		1,483	98.15	28	1.85	
Triage level							< 0.001					0.129					0.966
Level 1	5,132	24.85	4,058	79.07	1,074	20.93		5,073	98.85	59	1.15		5,074	98.87	58	1.13	
Level 2	11,623	56.29	10,777	92.72	846	7.28		11,505	98.98	118	1.02		11,483	98.80	140	1.20	
Level 3	3,625	17.56	3,292	90.81	333	9.19		3,573	98.57	52	1.43		3,580	98.76	45	1.24	
Level 4 and 5	268	1.30	240	89.55	28	10.45		263	98.13	5	1.87		265	98.88	3	1.12	
PCI							< 0.001					< 0.001					< 0.001
No	3,317	16.06	2,167	65.33	1,150	34.67		3,213	96.86	104	3.14		3,243	97.77	74	2.23	
Yes	17,331	83.94	16,200	93.47	1,131	6.53		17,201	99.25	130	0.75		17,159	99.01	172	0.99	
Hospital level							< 0.001					< 0.001					0.004
Medical centers	6,831	33.08	6,283	91.98	548	8.02		6,797	99.50	34	0.50		6,774	99.17	57	0.83	
Regional hospitals	11,305	54.75	10,021	88.64	1,284	11.36		11,172	98.82	133	1.18		11,150	98.63	155	1.37	
District hospitals	2,512	12.17	2,063	82.13	449	17.87		2,445	97.33	67	2.67		2,478	98.65	34	1.35	
Hospital ownership							< 0.001					0.025					0.486
Public	6,291	30.47	5,511	87.60	780	12.40		6,204	98.62	87	1.38		6,211	98.73	80	1.27	
Private	14,357	69.53	12,856	89.55	1,501	10.45		14,210	98.98	147	1.02		14,191	98.84	166	1.16	

A logistic regression model that applied GEEs was employed to estimate the effect of the 24-h PCI service model on the risk of death within 30 days after an emergency department visit among the patients with STEMI. Table 2 indicates that after the relevant variables were controlled, the risk of death among the patients with STEMI who were sent to hospitals with 24-h PCI services was significantly lower (lower by 15%) relative to that of the patients with STEMI who were sent to hospitals without 24-h PCI services (OR 0.85; 95% CI 0.75–0.98; *P* < 0.05).

**Table 2:** Relative risk of death within 30 days after an emergency department visit among the patients with STEMI.

Variables	Death within 30 days				
	Adjusted OR	95% CI			*p*-value
24-h PCI model					
No (ref.)	1				
Yes	0.85	0.75	–	0.98	0.020
Gender					
Female (ref.)	1				
Male	0.82	0.73	–	0.91	< 0.001
Age (years)					
< 45 (ref.)	1				
45–54	1.25	0.94	–	1.66	0.121
55–64	1.53	1.17	–	1.99	0.002
65–74	2.64	2.03	–	3.45	< 0.001
75–84	4.51	3.46	–	5.88	< 0.001
≧ 85	7.75	5.87	–	10.22	< 0.001
CCI score					
0 (ref.)	1				
1	1.30	1.13	–	1.49	< 0.001
2	1.45	1.23	–	1.71	< 0.001
3	1.39	1.16	–	1.67	< 0.001
≧ 4	1.67	1.42	–	1.96	< 0.001
Monthly salary					
≦ 17,280 (ref.)	1				
17,281–22,080	0.89	0.77	–	1.04	0.149
22,081–36,300	0.81	0.72	–	0.92	0.001
≧ 36,301	0.78	0.67	–	0.91	0.001
Urbanization level					
Level 1 (ref.)	1				
Level 2	1.07	0.92	–	1.24	0.388
Level 3	0.92	0.78	–	1.09	0.337
Level 4	1.06	0.90	–	1.25	0.495
Level 5–7	1.19	1.00	–	1.42	0.056
Other catastrophic illness					
No (ref.)	1				
Yes	1.53	1.30	–	1.80	< 0.001
Triage level					
Level 1 (ref.)	1				
Level 2	0.34	0.30	–	0.38	< 0.001
Level 3	0.31	0.27	–	0.36	< 0.001
Level 4 and 5	0.28	0.18	–	0.44	< 0.001
PCI					
No (ref.)	1				
Yes	0.20	0.18	–	0.22	< 0.001
Hospital level					
Medical centers (ref.)	1				
Regional hospitals	1.19	1.06	–	1.34	0.005
District hospitals	1.43	1.21	–	1.69	< 0.001
Hospital ownership					
Public (ref.)	1				
Private	0.94	0.84	–	1.04	0.227

After the analysis of the logistic regression model that applied GEEs was completed, the effect of the 24-h PCI service model on the risk of emergency department revisits within 3 days after discharge among the patients with STEMI was explored. Table 3 reveals that after the relevant variables were controlled, the risk of emergency department revisits within 3 days after discharge among the patients with STEMI who were sent to hospitals with 24-h PCI services was lower than that among the patients with STEMI who were sent to hospitals without 24-h PCI services. However, the difference was nonsignificant (OR 0.73; 95% CI 0.48–1.10; *P* > 0.05).

**Table 3:** Relative risk of emergency department revisits within 3 days after discharge among the patients with STEMI.

Variables	Emergency department revisits within 3 days				
	Adjusted OR	95% CI			*p*-value
24-h PCI model					
No (ref.)	1				
Yes	0.73	0.48	–	1.10	0.133
Gender					
Female (ref.)	1				
Male	1.05	0.77	–	1.45	0.752
Age (years)					
< 45 (ref.)	1				
45–54	1.30	0.68	–	2.46	0.427
55–64	1.33	0.72	–	2.47	0.360
65–74	1.36	0.72	–	2.56	0.351
75–84	1.71	0.90	–	3.25	0.101
≧ 85	2.13	1.08	–	4.17	0.028
CCI score					
0 (ref.)	1				
1	1.30	0.91	–	1.87	0.153
2	1.50	0.98	–	2.32	0.065
3	1.27	0.77	–	2.08	0.347
≧ 4	1.30	0.83	–	2.04	0.247
Monthly salary					
≦ 17,280 (ref.)	1				
17,281–22,080	0.69	0.45	–	1.06	0.093
22,081–36,300	0.90	0.65	–	1.25	0.534
≧ 36,301	1.11	0.75	–	1.62	0.610
Urbanization level					
Level 1 (ref.)	1				
Level 2	1.16	0.76	–	1.77	0.490
Level 3	1.33	0.84	–	2.11	0.222
Level 4	1.36	0.86	–	2.16	0.193
Level 5–7	1.91	1.20	–	3.06	0.007
Other catastrophic illness					
No (ref.)	1				
Yes	1.43	0.93	–	2.20	0.101
Triage level					
Level 1 (ref.)	1				
Level 2	1.12	0.81	–	1.54	0.490
Level 3	1.14	0.78	–	1.67	0.510
Level 4 and 5	1.19	0.47	–	3.02	0.720
PCI					
No (ref.)	1				
Yes	0.33	0.24	–	0.43	< 0.001
Hospital level					
Medical centers (ref.)	1				
Regional hospitals	1.94	1.32	–	2.87	< 0.001
District hospitals	3.09	1.96	–	4.87	< 0.001
Hospital ownership					
Public (ref.)	1				
Private	0.82	0.62	–	1.08	0.149

After the analysis of the logistic regression model that applied GEEs was completed, the effect of the 24-h PCI service model on the risk of readmission within 14 days after discharge from an emergency department among the patients with STEMI was estimated. Table 4 reveals that after the relevant variables were controlled, the risk of readmission within 14 days after discharge from an emergency department among the patients with STEMI who were sent to hospitals with 24-h PCI services was lower than that among the patients with STEMI who were sent to hospitals without 24-h PCI services. Nevertheless, the difference was nonsignificant (OR 0.87; 95% CI 0.62–1.22; *P* > 0.05). Furthermore, we conducted an analysis, as shown in Supplementary Table S1, that only focused on STEMI patients with PCI to compare the differences between hospitals providing 24-h PCI services or not. After the relevant variables were controlled, the risk of death after an ED visit among the patients with STEMI undergoing PCI who were sent to hospitals with 24-h PCI services was significantly lower than that among the patients with STEMI undergoing PCI who were sent to hospitals without 24-h PCI services (OR 0.83; 95% CI 0.71–0.97). Among STEMI patients with PCI, a similar result was obtained.

**Table 4:** Relative risk of readmission within 14 days after discharge from an emergency department among the patients with STEMI.

Variables	Readmission within 14 days				
	Adjusted OR	95% CI			*p*-value
24-h PCI model					
No (ref.)	1				
Yes	0.87	0.62	–	1.22	0.409
Gender					
Female (ref.)	1				
Male	1.21	0.87	–	1.69	0.255
Age (years)					
< 45 (ref.)	1				
45–54	1.06	0.60	–	1.89	0.837
55–64	1.31	0.76	–	2.24	0.335
65–74	1.19	0.68	–	2.11	0.541
75–84	1.24	0.69	–	2.23	0.479
≧ 85	1.55	0.82	–	2.94	0.179
CCI score					
0 (ref.)	1				
1	1.36	0.96	–	1.91	0.084
2	1.35	0.87	–	2.11	0.179
3	1.34	0.82	–	2.19	0.247
≧ 4	1.94	1.28	–	2.95	0.002
Monthly salary					
≦ 17,280 (ref.)	1				
17,281–22,080	0.95	0.63	–	1.43	0.799
22,081–36,300	1.19	0.87	–	1.63	0.285
≧ 36,301	0.71	0.47	–	1.09	0.119
Urbanization level					
Level 1 (ref.)	1				
Level 2	2.10	1.33	–	3.29	0.001
Level 3	2.13	1.31	–	3.46	0.002
Level 4	1.97	1.20	–	3.26	0.008
Level 5–7	2.22	1.32	–	3.73	0.003
Other catastrophic illness					
No (ref.)	1				
Yes	1.16	0.75	–	1.79	0.510
Triage level					
Level 1 (ref.)	1				
Level 2	1.23	0.90	–	1.69	0.185
Level 3	1.09	0.73	–	1.62	0.668
Level 4 and 5	0.93	0.29	–	3.01	0.904
PCI					
No (ref.)	1				
Yes	0.49	0.37	–	0.67	< 0.001
Hospital level					
Medical centers (ref.)	1				
Regional hospitals	1.44	1.05	–	1.97	0.022
District hospitals	1.12	0.71	–	1.78	0.619
Hospital ownership					
Public (ref.)	1				
Private	0.90	0.68	–	1.18	0.430

## Discussion

After the relevant variables were controlled, the risk of death among the patients with STEMI who were sent to hospitals with 24-h PCI services was significnately lower than that among the patients with STEMI who were sent to hostpials without 24-h PCI services. A reason may be that most hospitals that provided 24-h PCI services in Taiwan were medical centers or regional hospitals. We also discovered that for hospital level, the risk of death among the patients who were sent to district hospitals was significantly higher than that among the patients who were sent to medical centers. Furthermore, the patients who were sent to hospitals with 24-h PCI services were less prone to undergoing delayed PCI treatment because of referrals. Studies have revealed that the risk of death among patients with AMI is significantly correlated with the time between the onset of AMI and the unblocking of vessels during PCI (i.e., ischemic time) and the time between arrival at an emergency room and the unblocking of vessels during PCI (D2B or D2W).^11–13^.

We discovered that the risk of death within 30 days after an emergency visit was significantly lower among the patients with STEMI who were sent to medical centers than among those who were sent to other types of hospitals. A reason may be that, relative to other types of hospitals, medical centers typically exhibited greater compliance with the clinical guidelines for patients with STEMI. Literature findings indicate that hospitals that exhibit greater compliance with clinical guidelines contribute to a significantly lower risk of death among patients.^14^ We also discovered that the patients who underwent PCI had a significantly lower risk of death relative to those who did not undergo PCI. In Taiwan, patients with STEMI who cannot undergo PCI are either referred to other hospitals or treated with fibrinolytic therapy. Some patients were not suitable for PCI because of their highly critical condition (occlusion of all three coronary arteries). A study reported that the risk of death among patients with AMI who underwent PCI directly is lower than that among patients who underwent fibrinolytic therapy.^15^ In addition, if a patient is referred to another hospital because they cannot undergo PCI at the first hospital that they were admitted to, their PCI treatment is delayed, which further affects their prognosis.^16,17^ The three aforementioned conditions are likely to significantly reduce the risk of death among patients who undergo PCI.

However, the 24-h PCI service model could not reduce the risk of an emergency department revisit within 3 days after discharge and the risk of readmission within 14 days after discharge. With the implementation of the National Health Insurance program, accessibility to medical care became high in Taiwan. Patients with STEMI who are likely to undergo full treatment and be discharged in a stable condition are treated through conventional procedures. This is demonstrated by the low rates of emergency department revisits within 3 days after discharge (1.13%) and the low rates of readmission within 14 days after discharge (1.19%) (Table 1).

Accordingly, we recommend a reasonable expansion of the 24-h PCI service model. The results indicate that the 24-h PCI service model significantly reduced the risk of death within 30 days after an emergency visit among patients with STEMI. Thus, the government should establish the 24-h PCI service model in all administrative districts to reduce the risk of death among patients with STEMI.

There are several strengths in the present study. Taiwan’s National Health Insurance program provides insurance coverage to approximately 99.9% of its citizens. Therefore, the data from the National Health Insurance Research Database provided a sufficient representation of Taiwan’s population. National, full-population, and multiyear data were applied in the present study; hence, the statistics were reliable. In addition, **t**he factors that can affect the prognosis of patients with STEMI (e.g., risk of death, risk of revisiting the emergency department within 3 days after discharge, and risk of readmission within 14 days after discharge) were comprehensively explored in the present study. The present study also has several limitations. We performed a retrospective analysis by using secondary data obtained from the National Health Insurance Research Database. Because of the data constraints of the database, we could not study the health behaviors, the timing of their cardiac catheterization procedures, and the admission time of the participants. This was a limitation of the present study. Although Taiwan's National Health Insurance coverage is extensive, economic considerations may still be a primary factor in healthcare treatment. Despite medical guidelines’ recommendations, it is still true that not every patient with PCI received drug-eluting stent implantation because Taiwan’s NHI only partially covered the cost of drug-eluting stents. In future research, we also suggest further analyzing the impact of other factors, such as CABG, drug-eluting stents, and medication such as ACEI/ARB, antiplatelet agents, and beta-blockers.

## Conclusions

After the relevant variables were controlled, the risk of death within 30 days after an emergency department visit among the patients with STEMI who were sent to hospitals with 24-h PCI services was significantly lower than that among the patients with STEMI who were sent to hospitals without 24-h PCI services (OR 0.85; 95% CI 0.75–0.98). Therefore, the establishment of the 24-h PCI service model significantly reduced the risk of death within 30 days after an emergency department visit among the patients with STEMI. However, the model could not reduce the risk of emergency department revisits within 3 days after discharge and the risk of readmission within 14 days after discharge.

## Material and methods

### Data sources

The data analyzed in the present study were secondary data. Data for the period from 2010 to 2018 were retrieved from the National Health Insurance Research Database for analysis. We collected data pertaining to the claims of outpatients, emergency patients, and inpatients; death cause statistics; and major illness. These data were compiled, and those that corresponded to the relevant variables were selected for analysis. Since the patient identifications in the National Health Insurance Research Database have been scrambled random identification numbers for insured patients by the Taiwan government for academic research use, the informed consent was waived by the Research Ethics Committee of the Taichung Jen-Ai Hospital. The research was conducted in accordance with the 1964 Declaration of Helsinki and amendments and was approved by the institutional review board (IRB) of the Taichung Jen-Ai Hospital (IRB NO. 111-20), Taiwan.

### Study participants

**Figure 1:**
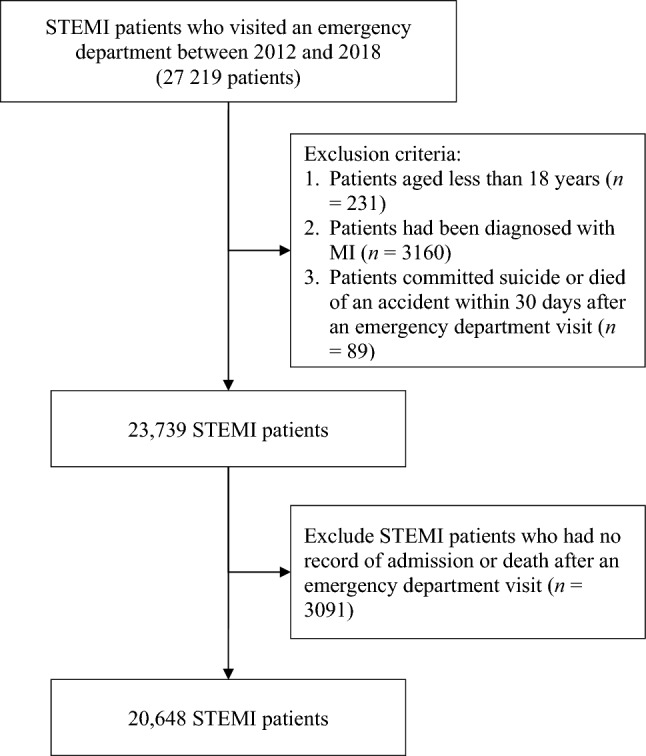
Flow chart of study participants.

We enrolled patients with STEMI who visited an emergency department between 2012 and 2018, and we analyzed whether the provision of 24-h PCI service affected the risk of death within 30 days after an emergency department visit, emergency department revisits within 3 days after discharge, and readmission within 14 days after discharge of patients with STEMI. Patients with STEMI who visited emergency departments were enrolled as participants; however, patients were excluded if they were aged less than 18 years, had been diagnosed with MI, committed suicide or died of an accident within 30 days after an emergency department visit, or had no record of admission or death after an emergency department visit. The emergency department visits were defined as medical behaviors that corresponded to the billing codes 00201B, 00202B, 00203B, 00204B, and 00225B. A diagnosis of STEMI was defined as a diagnosis made with the following codes: 410.0X–410.6X and 410.8X of ICD-9-CM or I21.01, I21.02, I21.09, I21.11, I21.19, I21.21, I21.29, I22.0, I22.1, I22.8, and I22.9 of ICD-10-CM. Figure 1 was the flow chart of study participants.

### Definition and description of variables

The definition of the variables considered in the present study are as follows. Twenty-four-hour PCI service was defined as a treatment performed in a hospital that provided 24-h PCI. A patient with STEMI was defined as a patient who was diagnosed with STEMI in accordance with the following principle discharge diagnosis codes: 410.0X–410.6X and 410.8X of ICD-9-CM or I21.01, I21.02, I21.09, I21.11, I21.19, I21.21, I21.29, I22.0, I22.1, I22.8, and I22.9 of ICD-10-CM. The gender of the patients was either male or female. The patients were divided into the following age groups: < 45 years, 45–54 years, 55–64 years, 65–74 years, 75–84 years, and ≥ 85 years. For monthly salary, the patients were divided into the following groups: ≤ NT$17 280, NT$17 281–NT$22 080, NT$22 081–NT$36 300, and ≥ NT$36 301. The urbanization levels of the patients’ residential areas were divided into Levels 1–7; Levels 1 and 7 indicated the highest and lowest levels of urbanization, respectively^18^. The hospitals investigated in the present study were classified as medical centers regional hospitals, and district hospitals. In Taiwan, hospitals are categorized into medical centers, regional hospitals, and district hospitals based on the results of hospital accreditation. Hospital accreditation includes many dimensions, such as the healthcare workforce, medical services provided, medical education, medical research, quality of medical care, etc. On the basis of ownership, the hospitals were classified as public and nonpublic hospitals. The comorbidity severity was estimated using Deyo’s Charlson Comorbidity Index (CCI). Deyo’s CCI is a modified version of the Charlson Comorbidity Index, and it classifies comorbidities into 17 types. The principal and secondary diagnosis codes of the patients were converted into weighted scores, which were then summed to calculate Deyo’s CCI scores^19^. The comorbidity severity was rated on a scale from 0 to 4 points. Classification of major illness comprised the presence (yes) and absence (no) of any major illness. The applied triage categories were Level 1 (Code 00201B), Level 2 (Code 00202B), Level 3 (Code 00203B), Level 4 (Code 00204B), and Level 5 (Code 00225B); Level 1 patients were those who most urgently required medical attention. Administration of PCI referred to patients who underwent PCI (codes 33076B, 33077B, and 33078B) during emergency visits or hospitalization.

### Quantitation of major results

In the present study, we primarily explored whether the 24-h PCI service model affected patients with STEMI with respect to their risk of death within 30 days after an emergency department visit, their emergency department revisits within 3 days after discharge, and their readmission within 14 days after discharge from an emergency department.

### Statistical analysis

The present study adopted a retrospective cohort design. SAS 9.4 (SAS Institute, Cary, NC, USA) was employed for secondary data processing and statistical analysis.

For descriptive statistics, the results pertaining to the following variables were expressed as percentages: age, gender, monthly salary, level of urbanization of residential area, severity of comorbidity (CCI), presence or absence of major illness, emergency triage level, performance of percutaneous coronary intervention (PCI), MI severity, death within 30 days after an emergency department visit, emergency department revisit within 3 days after discharge, readmission within 14 days after discharge, 24-h PCI capability of hospital, hospital level, and hospital ownership.

Inferential statistics were used to determine whether the 24-h PCI service model affected patients with STEMI in terms of risk of death within 30 days after an emergency department visit, emergency department revisits within 3 days after discharge, and readmission within 14 days after discharge from an emergency department. A chi-square test was first performed to analyze the differences between patients with MI who were sent to hospitals with 24-h PCI services and those who were sent to hospitals without 24-h PCI service in terms of mortality rate within 30 days after an emergency department visit, emergency department revisits within 3 days after discharge, and readmission within 14 days after discharge. Subsequently, a logistic regression model that employed generalized estimating equations (GEEs) was applied to compare differences between patients with STEMI who were sent to hospitals with 24-h PCI services and those who were sent to hospital without 24-h PCI services regarding the risk of death within 30 days after an emergency department visit, emergency department revisits within 3 days after discharge, and readmission within 14 days after discharge while controlling for other factors (i.e., gender, age, monthly salary, level of urbanization of residential area, CCI, presence or absence of major illness, hospital level, hospital ownership, and triage level). Besides, we conducted an analysis that only focused on STEMI patients with PCI to compare the differences between hospitals providing 24-h PCI services or not.

### Supplementary Information

Supplementary Table S1.

## Data Availability

The National Health Insurance Database used to support the findings of this study were provided by the Health and Welfare Data Science Center, Ministry of Health and Welfare (HWDC, MOHW) under license and so cannot be made freely available. Requests for access to these data should be made to HWDC (https://dep.mohw.gov.tw/dos/np-2497-113.html).
