# Supplementary material for: 24-h PCI model does affect the outcome of STEMI patients: a population-based study
Source: Sci Rep. 2023 Aug 11;13:13063. doi: 10.1038/s41598-023-40276-5 (PMC10421952; doi:10.1038/s41598-023-40276-5)
Supplement: Supplementary file 1 — Supplementary Table S1. [file 41598_2023_40276_MOESM1_ESM.docx]

Supplementary Table S1. Relative risks of death, emergency department revisits, and readmission in patients receiving PCI

| **Variables** | **Death**  **within 30 days** | | | | | **Emergency department revisits**  **within 3 days** | | | | | **Readmission**  **within 14 days** | | | | |
| --- | --- | --- | --- | --- | --- | --- | --- | --- | --- | --- | --- | --- | --- | --- | --- |
|  | **Adjusted**  **OR** | **95% CI** | | | **p-value** | **Adjusted**  **OR** | **95% CI** | | | **p-value** | **Adjusted**  **OR** | **95% CI** | | | **p-value** |
| 24-hour PCI model |  |  |  |  |  |  |  |  |  |  |  |  |  |  |  |
| No (ref.) | 1 |  |  |  |  | 1 |  |  |  |  | 1 |  |  |  |  |
| Yes | 0.83 | 0.71 | - | 0.97 | 0.020 | 0.98 | 0.63 | - | 1.51 | 0.909 | 0.78 | 0.53 | - | 1.15 | 0.210 |
| Gender |  |  |  |  |  |  |  |  |  |  |  |  |  |  |  |
| Female (ref.) | 1 |  |  |  |  | 1 |  |  |  |  | 1 |  |  |  |  |
| Male | 0.78 | 0.67 | - | 0.90 | <0.001 | 1.12 | 0.71 | - | 1.77 | 0.636 | 1.13 | 0.75 | - | 1.70 | 0.553 |
| Age (years) |  |  |  |  |  |  |  |  |  |  |  |  |  |  |  |
| <45 (ref.) | 1 |  |  |  |  | 1 |  |  |  |  | 1 |  |  |  |  |
| 45-54 | 1.00 | 0.70 | - | 1.43 | 0.996 | 1.65 | 0.71 | - | 3.84 | 0.242 | 0.99 | 0.50 | - | 1.98 | 0.976 |
| 55-64 | 1.45 | 1.04 | - | 2.02 | 0.031 | 1.60 | 0.70 | - | 3.65 | 0.263 | 1.42 | 0.75 | - | 2.69 | 0.283 |
| 65-74 | 2.43 | 1.75 | - | 3.39 | <0.001 | 1.28 | 0.53 | - | 3.09 | 0.578 | 1.41 | 0.72 | - | 2.75 | 0.312 |
| 75-84 | 4.72 | 3.40 | - | 6.57 | <0.001 | 2.39 | 1.01 | - | 5.67 | 0.048 | 1.68 | 0.84 | - | 3.35 | 0.143 |
| ≧85 | 9.14 | 6.45 | - | 12.94 | <0.001 | 4.65 | 1.88 | - | 11.52 | <0.001 | 1.98 | 0.88 | - | 4.43 | 0.097 |
| CCI score |  |  |  |  |  |  |  |  |  |  |  |  |  |  |  |
| 0 (ref.) | 1 |  |  |  |  | 1 |  |  |  |  | 1 |  |  |  |  |
| 1 | 1.34 | 1.12 | - | 1.60 | <0.001 | 1.42 | 0.90 | - | 2.26 | 0.134 | 1.44 | 0.97 | - | 2.15 | 0.073 |
| 2 | 1.56 | 1.26 | - | 1.92 | <0.001 | 1.72 | 0.99 | - | 3.01 | 0.056 | 1.49 | 0.89 | - | 2.49 | 0.125 |
| 3 | 1.49 | 1.17 | - | 1.88 | <0.001 | 1.44 | 0.74 | - | 2.82 | 0.284 | 1.82 | 1.06 | - | 3.14 | 0.031 |
| ≧4 | 1.93 | 1.56 | - | 2.38 | <0.001 | 1.54 | 0.82 | - | 2.88 | 0.178 | 1.73 | 1.01 | - | 2.96 | 0.044 |
| Monthly salary |  |  |  |  |  |  |  |  |  |  |  |  |  |  |  |
| ≦17,280 (ref.) | 1 |  |  |  |  | 1 |  |  |  |  | 1 |  |  |  |  |
| 17,281-22,080 | 0.90 | 0.74 | - | 1.09 | 0.288 | 0.95 | 0.54 | - | 1.70 | 0.874 | 0.99 | 0.60 | - | 1.64 | 0.966 |
| 22,081-36,300 | 0.74 | 0.63 | - | 0.87 | <0.001 | 0.97 | 0.62 | - | 1.53 | 0.890 | 1.28 | 0.87 | - | 1.88 | 0.209 |
| ≧36,301 | 0.75 | 0.61 | - | 0.91 | 0.004 | 1.29 | 0.77 | - | 2.17 | 0.334 | 0.80 | 0.48 | - | 1.33 | 0.392 |
| Urbanization level |  |  |  |  |  |  |  |  |  |  |  |  |  |  |  |
| Level 1 (ref.) | 1 |  |  |  |  | 1 |  |  |  |  | 1 |  |  |  |  |
| Level 2 | 1.09 | 0.90 | - | 1.32 | 0.369 | 1.68 | 0.89 | - | 3.19 | 0.112 | 2.15 | 1.23 | - | 3.77 | 0.007 |
| Level 3 | 0.96 | 0.77 | - | 1.19 | 0.702 | 2.11 | 1.08 | - | 4.11 | 0.029 | 2.25 | 1.24 | - | 4.07 | 0.007 |
| Level 4 | 1.06 | 0.85 | - | 1.32 | 0.626 | 2.51 | 1.28 | - | 4.93 | 0.008 | 1.97 | 1.06 | - | 3.67 | 0.033 |
| Level 5-7 | 1.18 | 0.94 | - | 1.49 | 0.143 | 2.50 | 1.23 | - | 5.06 | 0.011 | 2.24 | 1.19 | - | 4.20 | 0.012 |
| Other catastrophic illness |  |  |  |  |  |  |  |  |  |  |  |  |  |  |  |
| No (ref.) | 1 |  |  |  |  | 1 |  |  |  |  | 1 |  |  |  |  |
| Yes | 1.58 | 1.28 | - | 1.95 | <0.001 | 1.35 | 0.72 | - | 2.55 | 0.351 | 1.16 | 0.66 | - | 2.04 | 0.617 |
| Triage level |  |  |  |  |  |  |  |  |  |  |  |  |  |  |  |
| Level 1 (ref.) | 1 |  |  |  |  | 1 |  |  |  |  | 1 |  |  |  |  |
| Level 2 | 0.37 | 0.32 | - | 0.42 | <0.001 | 0.94 | 0.62 | - | 1.43 | 0.788 | 0.98 | 0.69 | - | 1.40 | 0.916 |
| Level 3 | 0.37 | 0.31 | - | 0.45 | <0.001 | 1.01 | 0.59 | - | 1.71 | 0.976 | 0.72 | 0.43 | - | 1.19 | 0.198 |
| Level 4 & 5 | 0.44 | 0.24 | - | 0.81 | 0.008 | - |  | - |  | - | 0.92 | 0.22 | - | 3.84 | 0.904 |
| Hospital level |  |  |  |  |  |  |  |  |  |  |  |  |  |  |  |
| Medical centers (ref.) | 1 |  |  |  |  | 1 |  |  |  |  | 1 |  |  |  |  |
| Regional hospitals | 1.22 | 1.06 | - | 1.42 | 0.007 | 1.76 | 1.12 | - | 2.75 | 0.014 | 1.33 | 0.93 | - | 1.91 | 0.119 |
| District hospitals | 1.19 | 0.94 | - | 1.51 | 0.143 | 2.13 | 1.14 | - | 3.95 | 0.017 | 1.42 | 0.83 | - | 2.43 | 0.200 |
| Hospital ownership |  |  |  |  |  |  |  |  |  |  |  |  |  |  |  |
| Public (ref.) | 1 |  |  |  |  | 1 |  |  |  |  | 1 |  |  |  |  |
| Private | 0.94 | 0.82 | - | 1.09 | 0.415 | 0.87 | 0.59 | - | 1.27 | 0.467 | 1.02 | 0.73 | - | 1.44 | 0.907 |
